# At the heart of mitochondrial quality control: many roads to the top

**DOI:** 10.1007/s00018-021-03772-3

**Published:** 2021-02-05

**Authors:** Roberta A. Gottlieb, Honit Piplani, Jon Sin, Savannah Sawaged, Syed M. Hamid, David J. Taylor, Juliana de Freitas Germano

**Affiliations:** Smidt Heart Institute, Cedars-Sinai Medical Center, AHSP9313, 127 S. San Vicente Blvd., Los Angeles, CA 90048 USA

**Keywords:** Mitochondria, Mitophagy, Fusion, Fission, Cardiac

## Abstract

Mitochondrial quality control depends upon selective elimination of damaged mitochondria, replacement by mitochondrial biogenesis, redistribution of mitochondrial components across the network by fusion, and segregation of damaged mitochondria by fission prior to mitophagy. In this review, we focus on mitochondrial dynamics (fusion/fission), mitophagy, and other mechanisms supporting mitochondrial quality control including maintenance of mtDNA and the mitochondrial unfolded protein response, particularly in the context of the heart.

## Introduction

Mitochondrial quality control plays a key role in long-lived cells including cardiomyocytes and neurons. Non-dividing cells require mechanisms to replace or repair proteins, complex assemblies of enzymatic machinery, and organelles, as well as membranes and potentially even DNA. In this review, we will focus on mitochondria, while acknowledging that repair/replacement mechanisms must also exist for other organelles including peroxisomes, lysosomes, endoplasmic reticulum, etc. While mitochondria are made up of protein assemblies, many of these are embedded in a lipid bilayer that requires ongoing maintenance, and uniquely, mitochondria are the only organelle with their own genome; thus, the integrity of mitochondrial DNA (mtDNA) must also be maintained. In this review, we will focus primarily on mitophagy. Mitochondrial quality control involves selective elimination of damaged mitochondria, replacement by mitochondrial biogenesis, redistributing newly imported proteins across the mitochondrial network by fusion, and segregation of damaged portions of the mitochondrial network by fission prior to mitophagy.

When the heart is stressed, mitochondria are frequently observed to change size and shape. The processes that mediate the fusion, fission and fragmentation of mitochondria comprise mitochondrial dynamics, and a competent mitochondrial dynamics system is essential for embryonic survival [1–3]. In general, mitochondria are regarded as existing on a binary spectrum between two extremes of fusion and fission. The common designations ‘elongated/tubular/fused’ and ‘fragmented/fissed’ are typically employed to describe these relative morphologic states, though more sophisticated descriptions also exist which incorporate interconnectedness along with size. The functional consequences of a fused mitochondrial network compared with a fragmented one are poorly understood; however, much of what is known about these morphologic states relates to how they interact with the mitochondrial quality control machinery.

## Molecular regulation of mitochondrial dynamics

**Fig. 1:**
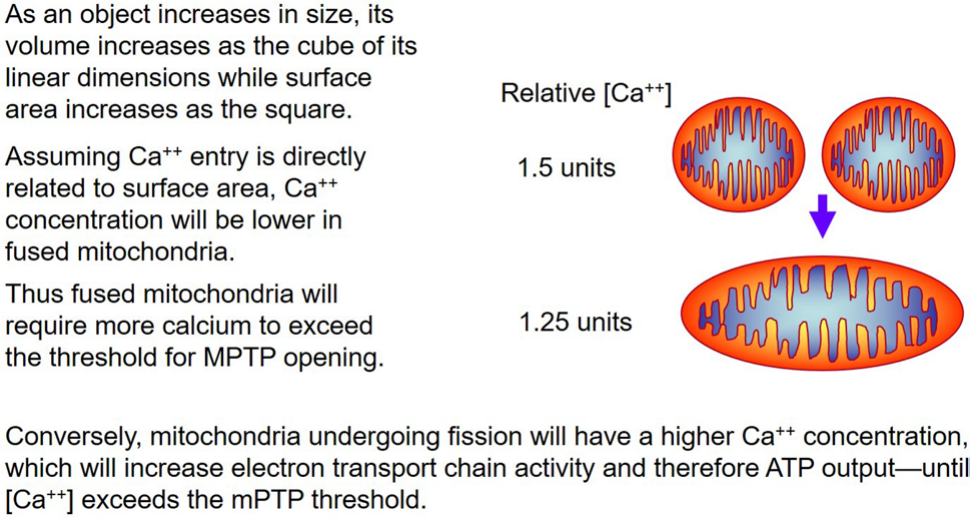
Surface area: volume relationship in mitochondria

The fusion/fission machinery is composed of dynamin-like GTPase proteins which reside either in the cytoplasm or on the inner (IMM) and outer mitochondrial membranes (OMM). Dynamin-related protein 1 (Drp1, or DNM1L), mainly located in the cytoplasm, is the principal effector of fission. Drp1 is activated by post-translational modifications and is recruited to mitochondria where it interacts with resident OMM receptors Mff, MiD49 and MiD51 [4]. Active Drp1 undergoes oligomerization, leading to membrane constriction with the process terminating with scission of the organelle mediated by Dynamin 2 [5]. Mitofusins 1 and 2 (Mfn1/2) are OMM resident GTPases, while Optic Atrophy 1 (Opa1) is located on the IMM. Collectively, they mediate mitochondrial fusion, though their regulation and function are incompletely understood. One reason for this discrepancy is that though a few target sites have been proposed, there are no established mechanisms of post-translational regulation of Mfn1/2 or Opa1, with the exception of proteolytic processing of Opa1. Moreover, the distinction between fusion and fission apparatus is not cut and dried since although Opa1 is recognized as a fusion protein, it frequently participates in fragmentation when proteolytic degradation alters its fusion activity [6]. One striking feature of mitochondrial dynamics is that mitochondrial networks can rapidly transition from a fused to a fragmented state in response to stress, while conditions that promote mitochondrial fusion lead to much slower morphologic changes. Acute mitochondrial fragmentation is frequently observed under conditions of increased energy demands, following either physiological or pathological stimuli in the exercising or ischemic heart, respectively [7, 8]. There are several theories which may explain why more active mitochondria undergo fragmentation. We have speculated that fragmentation may serve to increase surface area for nutrient and oxygen exchange, which will facilitate respiration—analogous to breaking up a long banquet table into smaller tables which increases the available seating. Alternatively, if membrane surface area is held constant during fragmentation, matrix volume will decrease, potentially elevating concentration of key solutes including Ca^+2^ (Fig. 1). Another intriguing hypothesis is that heat generated by active mitochondria poses a thermal stress to these organelles, and that by increasing the mitochondrial membrane surface area in contact with cytosol, this thermal energy can be more rapidly dissipated. Finally, there exists a large body of data to support a role for mitochondrial fission as a key step in the process of maintaining cellular homeostasis through the sequestration and subsequent elimination of damaged mitochondria by mitophagy.

The OMM allows selective transport of metabolites and connects the mitochondria to neighboring organelles. The IMM contains two separate compartments, the intermembrane space and the matrix, that are uniquely characterized by their cristae structures. The cristae provide the sites of the respiratory chain that are stabilized by the phospholipid cardiolipin (CL) whose IMM binding is required for the function of the ADP–ATP translocator, supercomplex stability, and cytochrome c oxidase [9, 10]. Under mitochondrial dysfunction, however, the CL pool undergoes significant change to alter mitochondrial dynamics and trigger mitophagy. CL exhibits further structural roles within the IMM to increase mitochondrial size: genetic mutation of the cardiac and skeletal muscle tafazzin results in 80% deficiency of CL synthesis and metabolism which is thought to contribute to cardiomyopathy in Barth Syndrome patients [11]. CL deficiency is further observed to cause cristae disorganization within cardiomyocytes and fibroblasts, ultimately giving way to decreased CL biosynthesis and initiation of mitophagy [12, 13]. In canonical mitophagy, CL serves as a signal for autophagic proteins to recognize dysfunctional mitochondria for degradation [14]. The mechanism of CL exposure from the IMM to OMM for cytosolic protein recognition remains in question, whether CL is translocated or perhaps externalized to the OMM during mitophagy.

One mechanism proposed for CL remodeling is the translocation of CL by phospholipid scramblases (PLS) that mediate translocation of phospholipids between the membrane bilayer. PLSCR3 is specifically enriched in the IMM and is important for maintenance of mitochondrial mass and respiration, yet is also required for sensitivity for cyt *c* release and CL mobilization [15]. UV irradiation nearly doubles the amount of CL on the OMM in a model of WT vs. mutant PLSCR3 HEK293 cells, suggesting that PLSCR3 facilitates CL transport. Another IMM protein NDPK-D has recently been demonstrated to facilitate CL mobilization upon mitochondrial uncoupling with CCCP in murine lung epithelial cells and human HeLa cells [16]. Interestingly, Kagan et al. discovered a novel interaction of NDPK-D with OPA1, implicating a close relationship of fission–fusion in PLS-mediated CL translocation.

Another proposed mechanism of exposure of CL is through OMM pores formed by Bax/tBid interaction [17, 18]. Evidence for membrane permeabilization by these apoptosis family member proteins comes from a combination of studies using mutagenesis [19], surface plasmon resonance [20], and glycosylation mapping [21] to determine the precise membrane-binding interactions. Specifically, during apoptosis, proteolysis of Bid by caspase-8 [22] or calpain [23] generates the active truncated form of Bid (tBid). The processed tBid interacts with Bax to promote the formation of pore-like structures in the OMM, enabling cytochrome *c* release from the IMM and potentially allowing cytosolic proteins to interact with CL on the inner membrane [24]. This can be amplified through a feedback cycle of CL interaction with procaspase-8 to facilitate processing to caspase-8 which can then process more Bid to tBid [25].

## Mitochondrial dynamics and mitochondrial quality control

For damaged mitochondria to be removed from the cell, they must be engulfed by the autophagosomal double-membrane structure as the phagophore is elongating. Mitochondrial dynamics and mitochondrial quality control are intimately linked in the heart: Several mitochondrial membrane fusion/fission proteins also interact with autophagy adaptors and effectors [26, 27]; furthermore, genetic ablation of fusion/fission proteins frequently leads to disordered mitochondrial autophagy [28–30].

While a clear picture of the contributions of fusion and fission to mitochondrial quality control has yet to emerge, it is generally assumed that during these processes, there is a sorting of mitochondrial components which segregates damaged proteins and organellar components away from the rest of the mitochondrial network. The concept of asymmetric mitochondrial fission was first described by the Shirihai group in β cells [31]. Dividing mitochondria were observed to exhibit different membrane potentials and those with higher membrane potentials were found to be more likely to fuse with other mitochondria. This of course begs the question of whether and how membrane potential could differ along the length of a mitochondrion prior to fission. That has recently been addressed by work [32] showing that individual mitochondrial cristae can have differing membrane potential; thus, it may be possible to have one region of a mitochondrion with high membrane potential (intact functioning individual cristae) and another region with low membrane potential (loss of cristae junctions and proton pumping capacity). After fission, mitochondria with lower membrane potential remained separate from the network and eventually are targeted for mitophagy, and thus, it was hypothesized that fission enables selection of mitochondria prior to autophagy. Healthy mitochondria would be capable of reintegration into the network, while damaged mitochondria would be unable to maintain adequate membrane potential and would subsequently be targeted for autophagic degradation. One mechanism for ensuring this is mediated by PINK1/Parkin, in which low membrane potential allows the accumulation of PINK1 on the outer membrane due to failure of protein import (which requires adequate membrane potential), and recruitment of Parkin, which ubiquitinates OM proteins. Ubiquitylation of Mfn1/Mfn2 prevents the mitochondrion from rejoining the network. It follows that during fusion, or in the interval between fission events, mitochondria sequester their healthy or damaged components to opposing poles of the mitochondria. Asymmetric fission of mitochondria has yet to be demonstrated in the intact heart, possibly in part due to the challenge of resolving mitochondria in adult cardiomyocytes using live microscopic techniques. Mitochondrial dynamism may operate differently in the heart than the evidence from cultured models suggests; some investigators have questioned the existence of networks of mitochondria in cardiac myocytes [33]. Recently however, Glancy et al. employed focused ion beam scanning electron microscopy to generate a three-dimensional model of cardiac mitochondria [34]. Their model proposed that multiple sub-networks of mitochondria exist in a cardiomyocyte, connected to the larger network via specific inter-mitochondrial junctions. Using live imaging, the authors further proposed that physical separation from the network occurs in malfunctioning mitochondria, leading to retraction of elongated mitochondria into condensed structures. It is reasonable to hypothesize that these separating mitochondria are undergoing fission, and we might, therefore, predict that there is increased mitochondrial autophagy activity in the electrically separated sub-networks. Recently, mitochondrial fusion events were demonstrated for the first time in adult ventricular cardiomyocytes [35]. Interestingly mitochondrial fusion rates decreased rapidly in culture in association with a decrease in calcium transient-driven contractile activity. Here, electrical uncoupling of the cardiomyocyte appeared to result in loss of fusion activity and in the previously mentioned Glancy report, electrical uncoupling of mitochondrial sub-networks had the same effect—suggesting that sustained cardiomyocyte–mitochondrial electrical coupling is important for maintaining mitochondrial fusion activity.

While there is evidence in cell models that piecemeal or bit-by-bit mitophagy is able to effectively sequester and degrade a portion of a mitochondrion, leaving the remaining organelle intact [36], it appears that the process of mitochondrial autophagy is generally impaired when mitochondria are more fused. In adult hearts lacking Drp1, mitochondria were elongated and dysfunctional [29]. Our unpublished data affirm this finding: dominant negative Drp1 suppressed basal autophagy and that induced by simulated ischemia/reperfusion (sI/R) in HL-1 cells, while wild-type Drp1 overexpression increased autophagy and decreased apoptosis in response to sI/R injury [Anne Hamacher-Brady and Roberta Gottlieb, unpublished data]. In Drp1-null hearts, mitochondria-associated p62 was increased, suggesting that while the initiation of mitophagy was enhanced, autophagic flux was impaired, and further in vitro studies using shRNA to silence Drp1 indicated that mitochondrial translocation to lysosomes was impaired even under basal conditions [29]. It is intuitive that there will be a greater cost to the cell to manufacture an autophagosome to encircle and engulf a larger fused mitochondrion than a smaller one; however, the relationship between fission and autophagy is likely more complex.

In addition to consideration of fission, it is important to understand the importance of fusion in mitochondrial quality control. Global constitutive knockout of both mitofusins results in embryonic lethality [3]. When both mitofusins are conditionally deleted in the heart, fusion activity is ablated, mitochondria appear smaller, and exhibit impaired rates of oxygen consumption [37]. Disruption of mitochondrial fusion in skeletal muscle through conditional deletion of Mfn1/2 (MLC1f promoter) results in accumulation of mtDNA point mutations and deletions and mtDNA depletion, resulting in muscle atrophy and impaired function [38]. Interestingly, the resulting energy deficit is accompanied by proliferation of small mitochondria with profoundly reduced mtDNA content (250 copies of mtDNA per nuclear genome in double KO vs 3500 copies in wild-type). Cardiac-restricted deletion of Mfn1/2 results in a similar proliferation of abnormal-appearing mitochondria, severe cardiomyopathy, and death by postnatal day 16 [39]. Hearts of these mice also exhibited mtDNA depletion. Interestingly, inducible cardiac-restricted deletion of Mfn1/2 exhibited mitochondrial dysfunction and poor cardiac contractility but a reduction in infarct size after acute I/R injury [40]. While disruption of fusion or fission individually is associated with significant mitochondrial dysfunction, the combined deletion of Mfn1/Mfn2 and Drp1 in the heart results in longer survival than either Mfn1/2 DKO or the Drp1 KO, but the mice eventually develop a unique hypertrophy associated with accumulation of mitochondria, impaired mitophagy, and mtDNA depletion [28].

## Canonical mitophagy

The best-characterized process for mitophagy is accomplished through the actions of PINK1/Parkin/P62. The genes encoding PINK1 and Parkin are strongly implicated as mediators of familial and sporadic Parkinson’s disease [41], and the first studies showing the interaction between these proteins in vivo identified a role of Parkin in restoring mitochondrial morphology and function downstream of PINK1 in mutant models of *Drosophila melanogaster* [42–44]. Further investigations confirmed PINK1–Parkin interactions in mammalian cells [45]. In this well-studied process, PINK1 is constitutively imported into the mitochondrion, where it is degraded by PARL in the intermembrane space [46] or Lon protease (LonP1) in the matrix [47].In the absence of adequate mitochondrial membrane potential PINK1 kinase is stabilized on the outer mitochondrial membrane [48]. As PINK1 accumulates, it phosphorylates multiple protein targets including ubiquitin [49, 50] [50, 51]; phosphoubiquitin activates and recruits Parkin, a cytosolic E3 ubiquitin ligase [50–54]. A recent study demonstrated that PINK1 also has a direct role in Parkin phosphorylation and subsequent activation [55]. Following Parkin activation and translocation, cytosolic p62 translocates to the mitochondria and binds to the polymerized ubiquitin through its ubiquitin-binding domain, and works as an adaptor connecting the damaged mitochondria to membrane of the autophagosomes through its LC3-interacting region (LIR), contributing to mitophagy completion [56]. PINK1 phosphorylation of ubiquitin and other targets can recruit NDP52 and optineurin to initiate Parkin-independent (but ubiquitin-dependent) mitophagy [57].

**Fig. 2:**
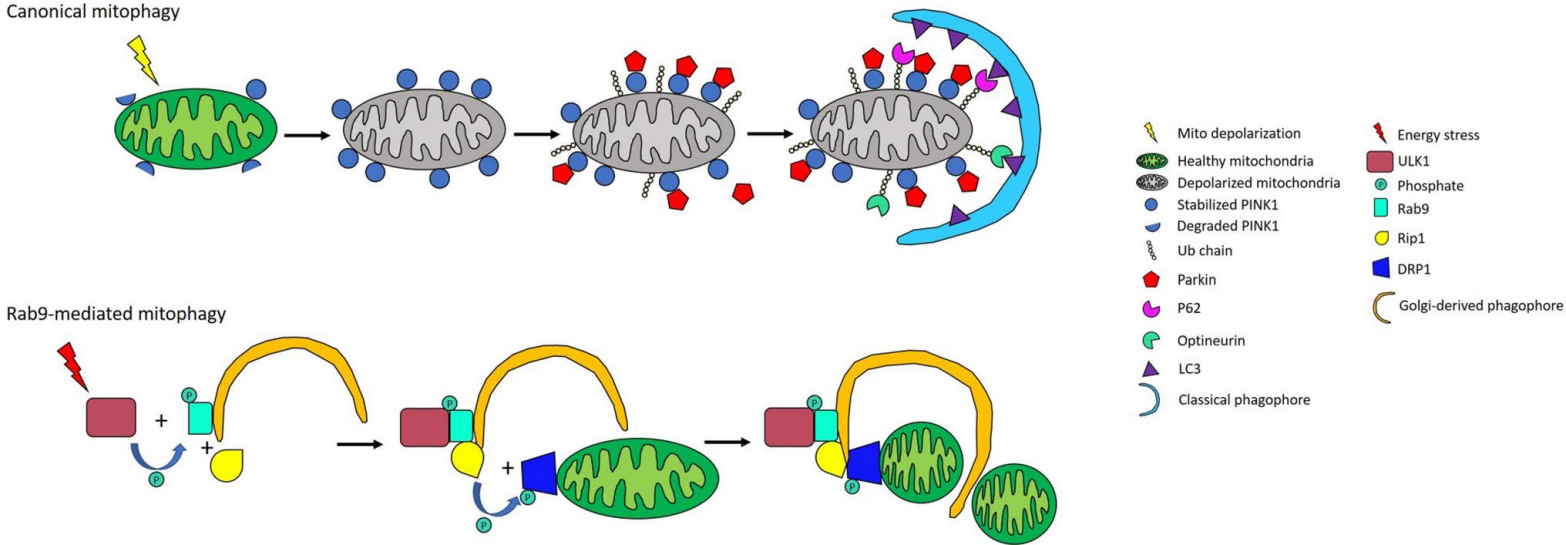
Canonical and alternative mitophagy pathways

Optineurin, first isolated by yeast two hybrid screening in 1998, is a 67-KDa protein implicated in many inflammatory conditions including cardiac ischemic disease. Optineurin is known to be involved in multiple processes of the cell such as autophagy [58], cell division [59], protein trafficking [60] and inflammatory signaling [61]. Optineurin can regulate mitophagy both through ubiquitin-dependent and -independent mechanisms. Optineurin interacts with LC3 through its LIR domain and this interaction is facilitated with phosphorylation by TBK1 at Ser177 [58]. In response to mitochondrial depolarization, TBK1 undergoes activating phosphorylation in a Parkin-PINK1 dependent manner [62], which facilitates optineurin recruitment to the damaged mitochondrion [57]. Following recruitment, optineurin binds with polyubiquitin chain through its Ub-binding domain (UBD) in ABIN proteins and NEMO (UBAN). Active TBK1 also phosphorylates optineurin at Ser473 and Ser513, stimulating the latter’s interaction with ubiquitin [63]. Optineurin also induces autophagosome formation by recruiting autophagy-associated proteins, namely the Atg12-5–16L1 complex [64]. In the ubiquitin-independent mechanism of optineurin, LC3 family members play an important role by recruiting optineurin to mitochondria by ubiquitin-like Atg8 protein. This further leads to a positive feedback loop mechanism of Atg8 lipidation [65]. We also recently documented the importance of optineurin in hypothermia-mediated cardioprotection [66]. Here, we showed that myocardial hypothermia applied after ischemia and reperfusion activated mitophagy and enhanced autophagic flux, reflected by downregulation of mitophagy markers including optineurin, parkin, and polyubiquitin chains in heavy membrane fraction. This event was accompanied by increased short form OPA1, MFF and DRP1. This process is summarized in Fig. 2.

Because the heart is a high-energy demanding organ, high-efficiency mitochondrial quality control preserves the functionality of the cardiomyocytes, and one would expect that PINK1/Parkin/p62 pathway would be involved in this regulation. Further experiments in mouse embryonic cardiomyocytes and fibroblasts revealed that Mfn2, an outer membrane fusion protein, gets phosphorylated by PINK1 and functions as a Parkin receptor [27]. Despite the existence of studies showing that Parkin-mediated mitophagy is dispensable for basal mitophagy in tissues of high metabolic demand, including the heart [67], further in vivo experiments supported the in vitro investigations regarding the role of Parkin in mitophagy. More specifically in cardiac disease, it was observed that Parkin accumulated in the border zone of wild-type infarcted mice but not in their Parkin-knockout (KO) counterparts, suggesting that Parkin is essential for mitophagy activation under myocardial stress [68]. Cardiac hypertrophy was also identified as a consequence of PINK1 knockout in mice, and PINK1 protein is drastically decreased in heart failure [69]. Further experiments revealed that cardiac hypertrophy as a consequence of diabetic cardiomyopathy induced by a high-fat diet was attenuated by but not exclusive of Parkin-mediated mitophagy, accompanied by attenuation of ventricular diastolic dysfunction [70]. Our group specifically demonstrated for the first time the critical role of Parkin/p62-mediated mitophagy in cardioprotection in an ischemic preconditioning model in mouse hearts [71]; subsequently, this mechanism was also validated in human atrial tissue during cardiopulmonary bypass heart surgery [72]. No evidence was found for the participation of Parkin in the clearance of mitochondria with damaged mtDNA in the cardiac aging process, as showed by Woodall et al. [73]. However, not only Parkin is essential for mitochondrial homeostasis during cardiac stress conditions but also it was found to be crucial for mitochondrial plasticity and metabolic reprogramming from carbohydrates to fatty acid oxidation in the perinatal mouse heart [74]. Moreover, Parkin was also implicated in the regulation of mitochondrial biogenesis in neurons through interaction with PGC-1α and ubiquitination of PINK1-phosphorylated PARIS (Parkin Interacting Substrate), which has a role in repressing PGC-1α. This mechanism, however, has not yet been analyzed in the heart. All these findings suggest that Parkin is a multifunctional protein with an essential role in maintaining mitochondrial quality control in different tissues through diverse mechanisms such as mitophagy, mitochondrial metabolic reprogramming and mitochondrial biogenesis.

A study by Hood’s group of age-related deterioration of skeletal muscle showed that exercise-induced mitophagy was mediated by Parkin, and that with aging, Parkin levels increased while mitochondrial respiration decreased [75]. The impaired respiration was worse in Parkin KO mice and was accompanied by increased ROS production in aged Parkin KO mice. Despite elevated Parkin protein in aged mice, its translocation to mitochondria after exercise was attenuated, in contrast to increased translocation in young mice. They also observed an inverse relationship between PGC-1α and PARIS, the repressor.

## Ubiquitin-independent mitophagy

In ubiquitin-independent mitophagy, autophagy receptors bind directly to dysfunctional mitochondria through the proteins present on the OMM, which link the dysfunctional mitochondria to autophagosomes. These receptors include Bnip3 (Bcl-2/adenovirus E1B interacting protein 3) and FUNDC1 present on OMM. These receptors contain one or more LIR (LC3-interacting region) [76] which bind to LC3 or GABARAP (gamma-aminobutyric acid receptor-associated protein) present on the phagophore. This interaction is regulated by phosphorylation status of the receptors; for example, Bnip3 phosphorylation on Serine residues 17 and 24 enhances binding to LC3B and GATE-16 [77]. Bnip3 is a hypoxia-inducible protein and a member of the proapoptotic Bcl-2 family, possessing a BH3 domain. Bnip3 integrates into the OMM via a carboxy terminal transmembrane (TM) domain and interacts with LC3 through the amino terminal domain [78]. Bnip3 was initially reported to induce cell death [79] through outer mitochondrial membrane permeabilization and cytochrome *c* release [80] as well as triggering the mitochondrial permeability transition pore (mPTP). Increasing evidence also implicate its role in cell survival by inducing autophagy [81, 82] and competition with Beclin1 for Bcl-2/Bcl-xL. Our group showed that Bnip3-mediated autophagy was mPTP independent [83]. Stress such as hypoxia induces mitochondrial quality by mitophagy through Bnip3 [82, 84]. Bnip3′s dual roles in cell fate suggest it is a pivotal regulator in disease processes [85, 86]. The Bnip3 promoter contains a consensus sequence for HIF-1α binding where it acts as transcription factor to drive Bnip3 expression [87]. One of the mechanisms shown to restrict Bnip3 expression in hypoxic conditions is methylation in the promoter region [88] which decreases the interaction between HIF-1α and the Bnip3 promoter. Chaanine et al. (2013) showed that Bnip3 knockdown prevented apoptosis, fibrosis, adverse cardiac remodeling and improved diastolic and systolic function in a heart failure model [89]. Overexpression of Bnip3 induces mitochondrial fragmentation in cardiomyocytes. Bnip3 has also been shown to interact with VDAC resulting in oligomerization of VDAC and mitochondrial dysfunction [89]. However, induced overexpression of Bnip3 did not lead to cardiomyocyte apoptosis in neonatal mice hearts [90]. Increasing reports support Bnip3′s dual roles to induce apoptosis and mitophagy; while phosphorylation may be one mechanism, other as yet unidentified processes may also regulate Bnip3′s prosurvival/proapoptotic functions.

Another mitophagy adapter protein, FUNDC1, is also shown to interact with LC3 to induce mitophagy in response to hypoxia [91]. FUNDC1 maintains the mitochondria-ER contact sites and promotes mitochondrial calcium uptake [92]. FUNDC1 interaction with LC3 is enhanced by phosphorylation at serine 17 by ULK1; mitochondrial phosphoglycerate mutase, PGAM5, dephosphorylates FUNDC1 [93, 94]. FUNDC1 knockout mice showed cardiac dysfunction and increased mitochondrial fission and cell death [95]. FUNDC1 interacts with Opa1, and its dephosphorylation promotes dissociation, leading to mitochondrial fission via interaction with DNM1L and culminating with mitophagy [96]. Many studies suggest the importance of FUNDC1 in regulating mitochondrial quality control and cardiac injury [97–99]; however, the detailed mechanism and interacting partners of FUNDC1 remain to be elucidated.

As a highly energetic organ, the heart heavily relies on properly functioning mitochondria to maintain normal function. Cardiac insults such as ischemia not only lead to mitochondrial dysfunction, but also excessive reactive oxygen species production and cell death. As such, the heart has been shown to be equipped with a number of alternate modes of mitochondrial clearance. Aside from the more traditional and well-characterized PINK/Parkin-mediated mitophagy pathway, another mechanism was recently identified revolving around Ulk1, Rab9, Rip1 and Drp1. Saito et al. reported that in a mouse model of coronary artery ligation, mitophagy was briskly activated in the heart [100]. Interestingly, mice with ATG7 knocked-out maintained this elevation in mitophagy; whereas, mitophagy was blunted when Ulk1 was knocked out. In this setting, Ulk1 appeared to play a predominant role in mediating mitophagy and expectedly, the absence of Ulk1 also corresponded with increased infarct size. They went on to show that these Ulk1-mediated autophagosomes originate from previously reported *trans* Golgi membranes enriched with Rab9 rather than LC3. Indeed, inhibiting Golgi membranes with brefeldin A revealed that mitophagy mediated by Ulk1 and Rab9 was impaired by brefeldin A, whereas conventional Parkin-mediated autophagy was unaltered by the drug. The authors showed that Rip1 induced the activating phosphorylation of Drp1 at S616 which triggered mitochondrial fission. Ulk1 was suggested to phosphorylate Rab9 at S179 which supported Rab9 and Rip1 interactions resulting in Drp1 phosphorylation. The authors suggest that these four proteins form a complex which indicates how mitochondrial fragments become trafficked into Ulk1-mediated autophagosomes (Fig. 2). This pathway appears to be important for cardiac homeostasis in diabetic cardiomyopathy [70], wherein Parkin is downregulated [101].

One might wonder why multiple mitophagy pathways may exist in the heart. Because the heart is highly reliant on healthy high functioning mitochondria, mitochondrial quality control machinery is crucial for maintaining cellular homeostasis. Therefore, mammals may have developed multiple context-dependent but only partially redundant mitophagy mechanisms.

## Quality control of the mitochondrial genome

Disruption of mitochondrial dynamics consistently impacted mtDNA integrity; it is, therefore, essential to consider mtDNA in any discussion of mitochondrial quality control. As mtDNA is continuously exposed to reactive oxygen species as a byproduct of respiration (about 1–2% of electrons go to superoxide generation [102]), and because it is not shielded by histones, it is vulnerable to damage, making ongoing maintenance or culling of deleterious mtDNA an essential element of cellular homeostasis. Mitochondria contain multiple copies of circular double-stranded DNA (mtDNA), which encodes tRNAs (22), rRNAs (2), and polypeptides (13) essential for oxidative phosphorylation. As almost the entire sequence encodes proteins or structural RNAs, a mutation anywhere in the ~ 16,000-bp sequence is likely to have consequences. mtDNA is not encased in histones but is condensed into nucleoids with TFAM, which also plays a role in mtDNA replication and transcription [103].

mtDNA repair depends on polymerase gamma (which also is responsible for mtDNA replication) and 8-oxoguanine DNA glycosylase (mOGG1). Defects in mtDNA repair lead to multiple disorders including heart failure [104]. Recently, the DNA repair nuclease MRE11A was linked to mitochondrial dysfunction including release of mtDNA into the cytosol where it triggered inflammasome activation and pyroptosis [105]. Another enzyme, DNA2, which functions in removal of single strand DNA during mtDNA replication or Long Patch Base Excision Repair pathway, has been linked to familial and sporadic forms of mitochondrial myopathy [106]. Doubtless many other enzymes can affect mtDNA integrity and repair, and their impaired function may eventually culminate in heart failure. Defects in mtDNA maintenance have been reviewed recently [107].

The mitophagy/mito-biogenesis/fusion/fission program has been suggested to be able to compensate for mtDNA damage that results in impaired respiratory function, based on elegant mathematical modeling of the process [108]. A key aspect was the requirement for excluding depolarized mitochondria from participation in fusion events. Parkin ubiquitinates multiple outer membrane proteins including mitofusin 1 and 2 [109, 110], causing their degradation by the proteasomal system, thereby preventing subsequent fusion events, and simultaneously promoting mitophagy. With aging, mtDNA damage increases, but it is not completely established whether this is due to attenuated mitophagy (which decreases with age).

## Mitochondrial unfolded protein response

Proper folding of proteins is essential for cellular homeostasis as aggregation of newly synthesized or imported misfolded proteins. Protein misfolding results in loss of individual protein functionality but also affects multi-protein complexes, leading to deleterious consequences for the cell [111]. To efficiently regulate protein folding processes, cells have developed distinct but highly integrated quality control mechanisms in the cytosol, endoplasmic reticulum and mitochondria. Cytosolic response to misfolded proteins relies heavily on a battery of heat shock proteins (Hsp), especially Hsp70, which leads to reprogramming of the cellular transcription program [112]. Endoplasmic reticulum has three highly conserved regulators of the unfolded response which provide surveillance across the ER membrane. These regulators—IRE1, PERK and ATF6—are kept dormant by the binding of Bip in the absence of misfolded proteins. Detachment of Bip due to accumulation of unfolded proteins leads to the activation of ER unfolded protein response which not only rewires the transcriptional program to increase the folding capacity but also suppresses RNA translation and decreases protein degradation programs to decrease the folding load [113, 114].

Regulation of unfolded proteins in mitochondria poses further challenges, primarily because of distinct structural compartmentalization and secondarily since mitochondrial proteins derive from both nuclear and mitochondrial genomes. Subunits translated from nuclear and mitochondrial transcripts must be assembled into the larger oxphos complexes in proper stoichiometry. Any imbalance between nuclear and mitochondrial protein synthesis can result in the accumulation of unincorporated proteins that may aggregate. The mitochondrial unfolded response UPR^mt^ is a transcriptional stress response that is activated by multiple forms of mitochondrial dysfunction in any of these compartments and reprograms mitochondrial to nuclear communication [115, 116].

In worms, activating transcription factor 1 (ATFS-1) is a well-studied transcription factor that acts as a first responder of UPR^mt^ activation. In addition to a mitochondrial targeting sequence (MTS), it also has a nuclear localization signal (NLS). Under homeostatic conditions, ATFS-1 is imported into mitochondria where it is degraded by LON protease [117, 118]. When mitochondria are damaged, ATFS-1 is preferentially accumulated in nucleus and activates UPR^mt^. Thus, compartmentalization of ATFS-1 regulates its transcriptional activity, indicating that mitochondrial import machinery plays an important role in UPR^mt^ induction. Although there are similarities in UPR^mt^ activation between worms and mammals, this process is certainly more complex in mammals. Many studies have shown that key components of integrated stress response (ISR), namely CHOP, ATF4 and ATF5 are not only activated by multiple forms of mitochondrial stress but are also required for the proper induction of UPR^mt^ [119, 120]. Among these, ATF5 is of special interest as it has been proposed to be a mammalian ortholog of ATFS-1 based on the fact that it can rescue UPR^mt^ activation in the absence of ATFS-1 [121]. Moreover, ATF5 bears a mitochondrial targeting sequence (MTS) and its activity seems to be regulated by mitochondrial import like ATFS-1 [121]. Despite the fact that ISR effectors like CHOP and ATF4 are activated in canonical UPR^mt^ signaling, the action of these factors is highly specific as they do not induce Bip, an ER chaperone important in UPR^ER^ [119]. Activation of the targeted transcription program due to these transcription factors leads to an increase in mitochondrial chaperones, thereby increasing mitochondrial folding capacity.

Reduction of protein import into mitochondria and a decrease in translation are also part of the UPR^mt^, which have been well documented in C. *elegans* [117, 122]. This phenomenon has also been reported recently in mammalian cells under acute induction of UPR^mt^, resulting in degradation of MRP3 transcript and protein [120]. This translational aspect of the UPR^mt^ may act locally on a single damaged mitochondrion, bypassing the need to cross any cellular threshold levels; this may represent the first line defense against mitochondrial damage [123]. Activation of UPR^mt^ also leads to the activation of estrogen receptor alpha (ERα), which leads to proteasome activation to decrease the misfolded protein burden especially in the inter-membrane space (IMS) [124, 125]. This signaling also activates HTRA2, a protease in the intermembrane space (IMS), and nuclear respiratory factor 1 (NRF1), which is involved in mitochondrial biogenesis [126]. LON protease is a member of AAA + proteases (ATPases associated with a variety of cellular activities), a broad group of ATP dependent proteases implicated in UPR^mt^ and responsible for the degradation of misfolded proteins in the mitochondrial matrix [127–129]. Recently, there have been a couple of reports relating the UPR^ER^ to UPR^mt^. A critical regulator of ER unfolded response, PERK was shown to regulate mitochondrial morphology promoting mitochondrial hyper-fusion and inhibiting severe mitochondrial fragmentation under conditions of stress [130]. In another study, activation of ISR under lipid stress was shown to upregulate LONP1 resulting in increased mitochondrial ROS and inflammasome activation in macrophages [131]. Future studies will reveal more exciting aspects of inter-organelle communication especially between mitochondria and endoplasmic reticulum.

Maintenance of mitochondrial function is vital for cell survival and functioning. This becomes of even more importance in organs where energy demand is high and cellular turnover is low, key characteristics of cardiomyocytes. Alterations in mitochondrial function due to impairment of mitochondrial quality control mechanisms are among the major causes of cardiac senescence and aging [132]. Mitochondrial unfolded protein response provides a robust mechanism for mitochondrial quality control by improving the homeostasis and limiting the damage.

## Conclusion

While mitochondrial quality control may be unimportant for “disposable” cells, it is indispensable for long-lived cells such as cardiomyocytes and neurons, where defects in mitochondrial quality control lead to functional deficits as seen in Parkin disease and many forms of heart failure. Multiple pathways exist that may represent housekeeping processes versus responses to different types of cellular stress. It is increasingly clear that maintaining mitochondrial quality control is essential to preserving cardiac function, for without good mitochondrial function, the energy to support contraction will be jeopardized. Interventions to target mitochondrial turnover are likely to enrich the therapeutic arsenal for heart disease.
